# Combination analysis of *NOS3*, *ABCB1* and *IL23R* polymorphisms with alcohol-induced osteonecrosis of the femoral head risk in Chinese males

**DOI:** 10.18632/oncotarget.16809

**Published:** 2017-04-04

**Authors:** Yuan Wang, Xuejun Yang, Jianping Shi, Yan Zhao, Linlin Pan, Jinqiu Zhou, Guoqiang Wang, Jianzhong Wang

**Affiliations:** ^1^ Department of Orthopedics, the People's Hospital of Manzhouli City, Manzhouli 021400, Inner Mongolia, China; ^2^ Department of Orthopedics, the Second Affiliated Hospital, Inner Mongolia Medical University, Hohhot 010030, Inner Mongolia, China; ^3^ The College of Traditional Chinese Medicine, Inner Mongolia Medical University, Hohhot 010010, Inner Mongolia, China; ^4^ Department of Endocrine, the 253th Hospital of People's Liberation Army of China, Hohhot 010010, Inner Mongolia, China

**Keywords:** NOS3, ABCB1, IL23R, osteonecrosis of the femoral head, gene polymorphism

## Abstract

**Background:**

Common variants of multiple genes played a crucial role in osteonecrosis of the femoral head (ONFH) onset which was proved by many previous reports. We hypothesized that polymorphisms in *NOS3*, *ABCB1* and *IL23R* were related to individual differences in alcohol sensitivity and the development of alcohol-induced ONFH.

**Methods:**

In this case-control study, we evaluated 8 SNPs in three genes in the Chinese Han population including 355 male cases and 355 healthy male controls. These SNPs were genotyped by Sequenom MassARRAY RS1000. To identify their relationship with alcohol-induced ONFH susceptibility using χ^2^ test and genetic model analysis.

**Results:**

We found an association with alcohol-induced ONFH susceptibility for 4 SNPs (rs743506, rs3918184, rs13233308 and rs6693831) in three genes after adjusted by age. The genotype “G/A” of rs743506 in *NOS3* gene acts as a risk factor in genotype (*P* = 0.003), dominant (*P* = 0.048), recessive (*P* = 0.005) and additive model(*P* = 0.006); The genotype “T/C” of rs3918184 in *NOS3* gene acts as a risk factor in genotype (*P* = 0.012) and recessive model (*P* = 0.009); The genotype “T/C” of rs13233308 in *ABCB1* gene acts as a risk factor in genotype (*P* = 0.038) and additive model(*P* = 0.041); The genotype “T/C” of rs6693831 in *IL23R* gene acts as a protective factor in genotype model (*P* = 0.046).

**Conclusions:**

This study provides evidence for three alcohol-induced ONFH susceptibility genes (*NOS3*, *ABCB1* and *IL23R*) in Chinese males and polymorphisms of them may be associated with alcohol-induced ONFH risk.

## INTRODUCTION

Osteonecrosis of the femoral head (ONFH) indicates a debilitating disorder that the bone collapses, happening in a special anatomical site of femoral head and resulting in osteocyte death [1, 2]. It mainly affects younger males who is between 30 and 50 years old. In China, there are seven million patients with osteonecrosis and be found annually new patients to increase by 150,000 to 200,000 patients [3]. The seventy-five to ninety percent cases of ONFH have been associated with various risk factors, involving alcohol abuse, corticosteroid use, hip trauma and smoking [4]. Increasing of adipose vesicles in the blood circulating is induced these risk factors, adding lipid deposition in osteocytes of the femoral head and resulting in embolism, which influence finally blood flow [5].

Current evidence suggests that being related to the risk of ONFH and eight SNPs in three genes of our research can be identified through the genome-wide association studies [1, 6, 7]. Nitric oxide is synthesised from L-arginine and has three isoforms of synthases (NOS): endothelial (eNOS) neuronal (nNOS) and inducible(iNOS). The eNOS is expressed in normal adult bone as a constitutive isoform [8]. A previous study in Korean patients indicated that idiopathic osteonecrosis of the femoral head was significantly associated with polymorphism of *eNOS* gene, but it also indicate that polymorphism in intron 4 of eNOS was not significant differences [9]. Glueck et al. found that the pathogenesis of idiopathic ONFH are related with the intron 4 of eNOS polymorphism and synthetic loss of nitric oxide outcome in African American and Caucasian patients. The ABCB1, is also called adenosine triphosphate-binding cassette B1, can encode the transport protein and made an significant influence on distribution and absorption in the mammalian body [10]. So far a few single nucleotide polymorphisms (SNPs) of *ABCB1* gene have been identified, of which mutations in exon 21 (G2677T) and exon 26 (C3435T) are associated with alteration of P-gp expression or function, as recently reviewed by Sakaeda et al. and Fromm et al [11, 12]. Interleukin 23 receptor (IL23) is a proinflammatory cytokine. According to previous reports indicated it inhibited osteoclastogenesis by Receptor Activator for Nuclear Factor κB Ligand with T cell affection [13]. Kim et al. discover polymorphisms in the Interleukin 23 receptor(*IL23R*) gene are associated with ONFH in Korean population [14].

A few studies investigated the association of *NOS3*, *ABCB1* and *IL23R* polymorphism with the pathogenesis of ONFH. We hypothesized that *NOS3*, *ABCB1* and *IL23R* genes have crucial roles in the development of alcohol-induced ONFH. To identify whether polymorphisms of these genes to be associated with alcohol-induced ONFH in Chinese males.

## RESULTS

In the current study, a total of 355 male cases (median age at diagnosis 44.91 ± 9.85 years) and 355 healthy male controls (median age 46.02 ± 9.61 years) were included. The basic characteristics of cases and controls were showed in Table 1. The primers of the 8 candidate SNPs are revealed in Table 2. Eight SNP loci(rs6693831, rs790631, rs4148749, rs10808072, rs13233308, rs3918227, rs3918184 and rs743506) in the *NOS3, ABCB1* and *IL23R* were evaluated in this study. SNP ID, HWE P value, allele A/B, MAF control/case, odds ratio, 95 % confidence interval and P value were listed in Table 3. Using x^2^ test, we found significant differences in frequency of alleles and rs13233308 in the *ABCB1* gene and rs743506 in the *NOS3* gene were associated with increased risk of alcohol-induced ONFH risk by allele model analysis (rs13233308, *P* =0.032, odds ratio [OR]: 1.26, 95% confidence interval [CI]: 1.02-1.55 and rs743506, *P* = 0.006, OR: 1.39, 95%CI: 1.09-1.78). All of the SNPs were in Hardy–Weinberg equilibrium (HWE) in the control population of this study.

**Table 1:** Characteristics of 355 alcohol-induced ONFH male subjects

Parameters	Cases	Controls	P value
Age [mean±SD]	44.91 ± 9.85	46.02 ± 9.61	NS
Sex [male]	355	355	
BMI (kg/m^2^)			
≥25	53	61	NS
< 25	302	294	NS

SD, standard deviation: NS, notsignificant: BMI, body mass index.

**Table 2:** Polymerase chain reaction primers of selected SNPs

SNP ID	1st – PCR primer sequences	2nd – PCR primer sequences	UEP sequences
rs6693831	ACGTTGGATGGTTACGGTCACCTTGGAAAG	ACGTTGGATGCTCAT AACCAGAAGATTCCC	TTCCCATGTGGGAAAGTTC
rs790631	ACGTTGGATGGTCTTATTAGGATAAAACCCC	ACGTTGGATGCCTTTT GCATATGCAGAAT	CACAAATACAATTCTCAAGTC
rs4148749	ACGTTGGATGACAGTCCCACTTGGATAAAG	ACGTTGGATGACAGAT GACACCACTTGGAG	attACACCACTTGGAGACCATATTTA
rs10808072	ACGTTGGATGCCTTTGTAACTTTCCCAATG	ACGTTGGATGCCTGAA AATGTTGTGTGTTGG	ggggTTGAGAATTGTATTGCTAGTTA
rs13233308	ACGTTGGATGCTGTTTCAATGATCCAGGTG	ACGTTGGATGGTTG GTGCTACCCTCAAAAT	cttcGCTACCCTCAAAATATATCCA
rs3918227	ACGTTGGATGTGAGTGCCGTTCATTGTGTG	ACGTTGGATGTTCAT AATAGCCCCGACCTG	gGTCACCAACAAGAGAATG
rs3918184	ACGTTGGATGCCATCGAGAAACATTACCCG	ACGTTGGATGCTTGA ATCCCTGACCTCAGC	gggagTACAGGCGTGAGCCACCA
rs743506	ACGTTGGATGGAGCAAGC TAGATTGCTAGG	ACGTTGGATGAAATG CACCCCCACCAAAAG	tcccgCCCTCTGGGCTCCTCTCC

PCR, polymorphism chain reaction; SNP, single-nucleotide polymorphism; UEP, unextended mini-sequencing primer.

**Table 3:** Allele frequencies of polymorphic variants of the candidate genes in this study

SNP ID	Chromosome	Position	Gene	HWE *P* value	Alleles A/B	MAF control	MAF case	Allele model	
								OR (95% CI)	*P* value
rs6693831	1	67720867	*IL23R*	0.1227	T/C	0.254	0.238	0.91(0.72-1.17)	0.48
rs790631	1	67676922	*IL23R*	0.5857	C/T	0.049	0.061	1.24(0.79-1.96)	0.35
rs4148749	7	87144413	*ABCB1*	0.5719	C/G	0.17	0.163	0.95(0.72-1.26)	0.72
rs10808072	7	87176463	*ABCB1*	0.4747	G/A	0.341	0.338	0.99(0.79-1.23)	0.91
rs13233308	7	87244960	*ABCB1*	0.9156	T/C	0.497	0.554	1.26(1.02-1.55)	0.032*
rs3918227	7	150700946	*NOS3*	0.6408	A/C	0.063	0.063	1(0.65-1.53)	1
rs3918184	7	150702219	*NOS3*	0.7983	T/C	0.292	0.339	1.25(0.99-1.56)	0.05
rs743506	7	150706915	*NOS3*	0.6418	G/A	0.218	0.280	1.39(1.09-1.78)	0.006*

OR, odds ratio, 95% CI, 95% confidence interval. *P < 0.05, statistical significance.

A/B stands for minor/major alleles on the control sample frequencies.

SNPs are excluded at HWE *P* < 0.05.

In this study, the whole of patients were male and we then further analyzed the association of the *NOS3*, *ABCB1* and *IL23R* gene polymorphisms with alcohol-induced ONFH patients adjusted by age in Table 4. The “G/A” of rs743506 increased alcohol-induced ONFH risk was found by the genotype model (*P* = 0.004, OR, 2.57; 95% CI, 1.36-4.86 crude; *P* = 0.003, OR, 2.66; 95% CI, 1.40-5.05 adjusted by age), the dominant model (*P* = 0.048, OR, 1.35; 95% CI, 1.01-1.82 adjusted by age), the recessive model (*P* = 0.006, OR, 2.40; 95% CI, 1.28-4.49 crude; *P* = 0.005, OR, 2.48; 95% CI, 1.32-4.65 adjusted by age) and the additive model (*P* = 0.008, OR, 1.38; 95% CI, 1.09-1.75 crude; *P* = 0.006, OR, 1.39; 95% CI, 1.10-1.78 adjusted by age), analysis respectively. The “T/C” of rs3918184 increased alcohol-induced ONFH risk was found by the genotype model (*P* = 0.016, OR, 1.86; 95% CI, 1.12-3.08 crude; *P* = 0.012, OR, 1.91; 95% CI, 1.15-3.18 adjusted by age) and the recessive model (*P* = 0.013, OR, 1.85; 95% CI, 1.14-2.99 crude; *P* = 0.009, OR, 1.89; 95% CI, 1.17-3.08 adjusted by age), analysis respectively. The “T/C” of rs13233308 increased alcohol-induced ONFH risk was showed by the genotype model (*P* = 0.031, OR, 1.59; 95% CI, 1.04-2.43 crude; *P* = 0.038, OR, 1.57; 95% CI, 1.03-2.39 adjusted by age), the dominant model (*P* = 0.049, OR, 1.43; 95% CI, 1.00-2.04 crude) and the additive model (*P* = 0.033, OR, 1.26; 95% CI, 1.02-1.55 crude; *P* = 0.041, OR, 1.25; 95% CI, 1.01-1.54 adjusted by age), analysis respectively. Meanwhile, we also found the “T/C” of rs6693831 decrease alcohol - induced ONFH risk by the genotype model (*P* = 0.041, OR, 0.72; 95% CI, 0.53-0.98 crude; *P* = 0.046, OR, 0.73; 95% CI, 0.53-0.99 adjusted by age).

**Table 4:** Association between single-nucleotide polymorphisms and risk of alcohol-induced ONFH based on logistic test

SNP ID	Model	Genotype	OR (95% CI)	*p* value	†OR (95% CI)	†*p* value
rs6693831	Genotype	T/C	0.72(0.53-0.98)	0.041*	0.73(0.53-0.99)	0.046*
	Dominant	T/C	0.79(0.59-1.07	0.121	0.80(0.59-1.08)	0.150
	Recessive	T/C	1.57(0.83-2.94)	0.163	1.67(0.88-3.15)	0.120
	Additive	T/C	0.92(0.72-1.17)	0.493	0.93(0.73-1.19)	0.570
rs790631	Genotype	C/T	2.05(0.18-2.27)	0.560	1.96(0.17-2.18)	0.582
	Dominant	C/T	1.23(0.76-1.99)	0.393	1.26(0.77-2.03)	0.356
	Recessive	C/T	2.01(0.81-2.22)	0.571	1.92(0.17-2.13)	0.593
	Additive	C/T	1.24(0.78-1.94)	0.358	1.25(0.79-1.97)	0.328
rs4148749	Genotype	C/G	0.74(0.31-1.79)	0.511	0.74(0.31-1.78)	0.498
	Dominant	C/G	0.97(0.71-1.34)	0.871	0.98(0.71-1.35)	0.902
	Recessive	C/G	0.74(0.31-1.78)	0.507	0.73(0.31-1.77)	0.492
	Additive	C/G	0.95(0.72-1.25)	0.724	0.95(0.72-1.26)	0.745
rs10808072	Genotype	G/A	0.99(0.56-1.57)	0.812	0.96(0.57-1.61)	0.874
	Dominant	G/A	1.01(0.74-1.35)	0.983	1.01(0.74-1.35)	0.982
	Recessive	G/A	0.93(0.57-1.51)	0.774	0.95(0.58-1.55)	0.845
	Additive	G/A	0.98(0.78-1.23)	0.906	0.99(0.79-1.24)	0.941
rs13233308	Genotype	T/C	1.59(1.04-2.43)	0.031*	1.57(1.03-2.39)	0.038*
	Dominant	T/C	1.43(1.00-2.04)	0.049*	1.41(0.99-2.01)	0.060
	Recessive	T/C	1.29(0.93-1.81)	0.124	1.29(0.92-1.79)	0.140
	Additive	T/C	1.26(1.02-1.55)	0.033*	1.25(1.01-1.54)	0.041*
rs3918227	Genotype	A/C	1.00(0.17-7.14)	1.000	1.06(0.14-7.59)	0.955
	Dominant	A/C	1.00(0.63-1.57)	1.000	1.01(0.64-1.58)	0.971
	Recessive	A/C	1.00(0.14-7.13)	1.000	1.06(0.15-7.17)	0.955
	Additive	A/C	1.00(0.65-1.52)	1.000	1.01(0.66-1.54)	0.963
rs3918184	Genotype	T/C	1.86(1.12-3.08)	0.016*	1.91(1.15-3.18)	0.012*
	Dominant	T/C	1.15(0.86-1.55)	0.346	1.16(0.86-1.56)	0.322
	Recessive	T/C	1.85(1.14-2.99)	0.013*	1.89(1.17-3.08)	0.009*
	Additive	T/C	1.23(0.99-1.54)	0.061	1.25(0.99-1.55)	0.051
rs743506	Genotype	G/A	2.57(1.36-4.86)	0.004*	2.66(1.40-5.05)	0.003*
	Dominant	G/A	1.33(0.99-1.79)	0.058	1.35(1.01-1.82)	0.048*
	Recessive	G/A	2.40(1.28-4.49)	0.006*	2.48(1.32-4.65)	0.005*
	Additive	G/A	1.38(1.09-1.75)	0.008*	1.39(1.10-1.78)	0.006*

OR, odds ratio; 95% CI, 95% confidence interval;

*P < 0.05, statistical significance. †Adjusted by age.

**Figure 1:**
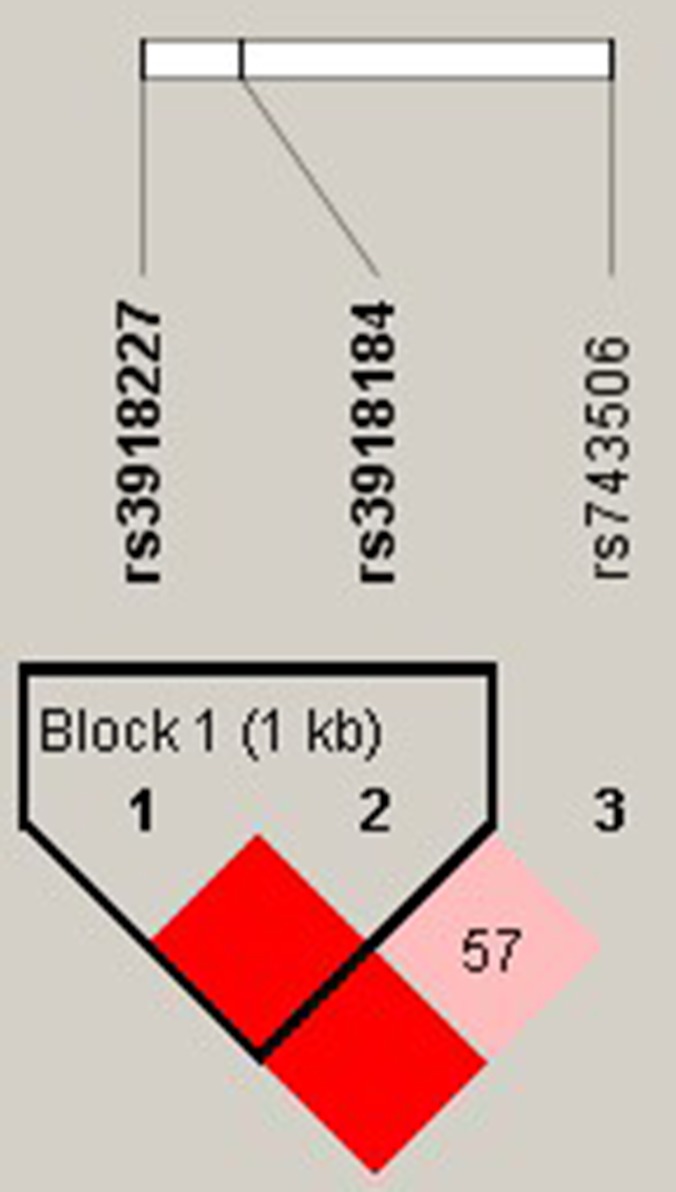
Haplotype-Block Map for *NOS3* Based on rs3918227, rs3918184 and rs743506 Linkage disequilibrium (LD) among single nucleotide polymorphisms (SNPs) analyzed in chromosome 7q. LD is indicated by standard color schemes with bright red for very strong LD (LD > 2, D’= 1) and pink red (LD > 2, D’< 1) for intermediate LD.

The results of the association between the *NOS3* haplotype and alcohol-induced ONFH risk were listed in Table 5. Haplotype “CT” in Block 1 was found to be associated with increased alcohol-induced ONFH risk (*P* = 0.044, OR, 1.26; 95% CI, 1.01-1.58 adjusted by age) (Figure 1).

**Table 5:** Haplotype analysis results of rs3918227 and rs3918184 in *NOS3* gene

Haplotype	freq(case)	freq(control)	†OR(95%CI)	†*P*
CT	0.340	0.293	1.26(1.01-1.58)	0.044*
AC	0.063	0.063	1.12(0.72-1.73)	0.610

Freq, frequency; OR, odd ratio; CI, confidence interval.

*P < 0.05, statistical significance: †Adjusted by age

## DISCUSSION

Non-traumatic ONFH has been diffusely awaked as a pathological define with numerous etiologies, the precise pathogenesis of osteonecrosis leaves to be evaluated. Alcoholism and chronic steroid use were considered to be the essential risk factors induced osteonecrosis. Genetic mutation of alcohol-metabolizing enzyme genes has been associated with alcohol-induced osteonecrosis and polymorphism of *NOS3*, *ABCB1* and *IL23R* gene are related with developing osteonecrosis [6, 15–17].

The *NOS3* gene located in chromosome 7q36.1 involves two SNPs (“G/A” in rs743506 and “T/C” in rs3918184) related with increased alcohol-induced ONFH risk. Endothelial nitric oxide synthase is the remarkable NOS isoform expressed in normal bone and eNOS adjusts bone resorption and formation and oestrogen are dependent on NO production via eNOS in the skeletal system [18]. Excessive NO production was found in various rheumatic diseases, including rheumatoid arthritis, systemic lupus erythematosus, osteoarthritis and vasculitis [8]. Güler S et al. found the genotype “GA” of rs743506 showed risk effect was associated with aura in migraine patients [19]. It proved that mutation of rs743506 would affect function of NO in cerebral blood flow regulation and include the activation of nociceptors in the vascular system and the release of vasoactive neuropeptides in the neurogenic inflammatory response [20]. Meanwhile, Juan P et al. found genotype “T/C” of rs3918184 be related with increased risk of hypertension, preeclampsia and stroke [21]. Combination with our research, rs743506 and rs3918184 associated with increased with alcohol-induced ONFH risk and it may also be effect NO in the femoral head blood flow regulation. The NOS is fundamentally expressed in vascular endothelium and had been proved to shorten NO norms in human plasma [22]. Our study suggests that the genotype “GA” of rs743506 and genotype “T/C” of rs3918184 polymorphisms may be risk factors for alcohol-induced ONFH, and NO brought in fundamentally decided eNOS may make a significant effect on the pathogenesis of alcohol-induced ONFH.

The *ABCB1* gene, also known as Multidrug-resistant transporter-1(*MDR1*), was located in chromosome 7q21.12 and the genotype “T/C” of rs13233308 related with increased alcohol-induced ONFH risk. This gene spans 4.5 kb encoding a 1280 amino acid (aa) polypeptide. After glycosylation of the amino acid polypeptide, a 170-kDa of protein named P-glycoprotein (P-gp) can be formed. P-gp is occurred in various tissue cells and is required for pumping lipophilic drugs (including glucocorticoids) out of the cells. This causes cytotoxic damage and the intracellular drug concentration to decrease is subsequently reduced. In addition, P-gp, encoded by the human *ABCB1* gene, is an important determinant in absorption, elimination of drugs and tissue targeting [23]. Xue et al. found polymorphisms of *ABCB1* gene be related with decreased risk of steroid-induced avascular necrosis of the femoral head in a Chinese population [16]. Zhang et al. also found *ABCB1* gene polymorphisms associated with decrease risk for steroid-induced osteonecrosis of the femoral head in Chinese population [1]. To date, 28 SNPs in the *ABCB1* gene have been reported at 27 positions in Caucasians and Africans [24, 25]. Apparently, these results was inconsistent with ours. Meanwhile, we must notice that ethnic differences may be considered to take an important effect to ABCB1 gene and ONFH. The homozygosity of both the wildtype and variant alleles of *MDR1* (C1236 T, G2677T/A, and C3435T) in healthy control subjects was similar to that reported by other Asian studies, which were performed with healthy Chinese, Japanese, and Korean subjects. However, the allele frequencies were significantly higher than those observed in European ethnicities, such as German, Russian, and Serbian [16]. Thus, further studies related with in other ONFH populations are required to confirm and realize *ABCB1* gene different mechanisms in steroid-induced and alcohol-induced ONFH.

The IL23R gene was located in chromosome 1p31.3 and the genotype “T/C” of rs6693831 related with decreased alcohol-induced ONFH risk. Interleukin 23 receptor, a heterodimer protein, was composed of two subunits: IL-23R and IL-12Rb1. IL-23R, expressed on activated T cells, dendritic cells, macrophages and natural killer (NK) cells, binds particularly to IL-23, produced primarily by dendritic cells and activated macrophages from peripheral tissues [26]. Kim et al. found polymorphisms of the *IL23R* gene were expressively related with ONFH among the Korean population. Among others, the polymorphism of the *IL23R* gene were particularly related with idiopathic ONFH risk. However, there was no significant associated with alcohol-induced and steroid-induced ONFH [14]. Apparently, this results were inconsistent compared with our results. In our study, rs6693831 of *IL23R* gene were related with decrease alcohol-induced ONFH risk in Chinese males. Alcoholism, which can influence fat metabolism and backlogging cell pressure, which considers absorption of the blood flood, were considerable risk factors with osteonecrosis [27]. We may thought whether the effect of alcohol abuse was stronger than genetic factors in regards to alcohol-induced ONFH patients. Meanwhile, the difference of results must be considered the effect related ethnic. In further studies, we need larger different ethnic populations to verify our results.

In this case–control study, some limitations were intrinsic and must be marked. First, to avoid selection bias, alcohol-induced ONFH cases and healthy controls came from the same hospital. However, this bias not necessarily to be of consequence because of demographic variables. Secondly, the sample size (355 male cases and 355 male controls) is not large enough in our work. We performed a power analysis and just found the power of rs743506 in *NOS3* gene was 0.91. The power of seven surplus SNPs was < 0.75. We considered that the number of samples was small in association studies and confirming our finding required a larger sample size [28].

Our research provides a new evidence for a relationship between *NOS3, ABCB1* and *IL23R* gene and alcohol-induced ONFH in Chinese males onset, which may shed light on the etiology of alcohol-induced ONFH. Functional studies are further required to evaluate the correlation of genotype and phenotype in a large cohort of various ethnicities.

## MATERIALS AND METHODS

### Ethics statement

The use of the protocol in this study was strictly affirmed to the principles expressed in the Declaration of Helsinki and were approved by the Ethical Committee of Zhengzhou Traditional Chinese Medicine Traumatology Hospital. Informed consent was signed from all of the participants.

### Study participants

Three hundred and fifty male patients with alcohol-induced ONFH were consecutively enrolled in this study at the Zhengzhou Traditional Chinese Medicine Traumatology Hospital from June 2014 to January 2016. Patients with a history of ethanol consumption of at least 400 ml per week were categorized under alcohol-induced osteonecrosis [29]. The development of alcohol-induced ONFH was diagnosed according to assessment by X-rays, magnetic resonance imaging (MRI), and bone scans [2]. The ONFH was present in one hip in 135 patients and in both hips in 220 patients (440 hips). Patients with a demonstrable history of direct trauma or with the possibility of a combination of causes were excluded.

We also enrolled 355 healthy male controls between July 2014 and January 2016 based on medical examination at Zhengzhou Traditional Chinese Medicine Traumatology Hospital. The controls had a history of ethanol consumption of at least 400 ml per week, however, they had no alcohol-induced ONFH and other related diseases, no history of thromboembolic events and no symptoms of hip disease. All participants were restricted to Chinese Han population who lived in Zhengzhou city and its surrounding areas.

### Genotyping

Genomic DNA was extracted and purified from the whole blood of all the participants using a kit (GoldMag, China) and their concentration was measured by spectrometry (DU530UV/VIS spectrophotometer; Beckman Instruments, Fullerton, CA). All PCR reactions were operated in a 25ul volume containing 10pmol of each primer, 1X PCR buffer, 10mM dNTP, 25 ng of genomic DNA, and 1U of Taq DNA polymerase (Solgent, Daejeon, Korea). The conditions of PCR amplification were as follows: initial denaturation at 95°C for 15 min followed by 35 cycles of denaturation at 95°C for 20 sec, annealing at 55°C for 40 sec, extension at 72°C for 1 min, and a final extension at 72°C for 5 min. SNP genotyping was performed on the SEQUENOM MassARRAY_Analyzer 4 (Sequenom, Inc., San Diego, CA, USA) using genomic DNA in a single multiplex reaction. According to the manufacturer's instructions, the primers for polymerase chain reaction amplification and single base extension were designed by Sequenom Assay Design 3.0 software (Sequenom, San Diego, CA, USA)[30]. SNP genotyping using the standard protocol recommended by the manufacturer was performed by Sequenom MassARRAY RS1000. Data analyses and management were conducted by Sequenom Typer 4.0 Software [30, 31].

### Statistical analysis

Microsoft Excel and SPSS 16.0 statistical package (SPSS, Chicago, IL) were used to perform statistical analyses. All *P* values in this study were two-sided, and *P* = 0.05 was considered the threshold of whether statistical significance was achieved or not. A significant departure of genotype frequency from Hardy–Weinberg equilibrium (HWE) for each SNP was estimated using SNPStats (http://bioinfo.iconcologia.net/snpstats/start.htm). The genotype frequencies of cases and controls were calculated using χ^2^ test [32]. Odds ratios (ORs) and 95% confidence intervals (95% CIs) were determined using unconditional logistic regression analysis with adjustment by age [33]. The four genetic models (Genotype, Dominant, Recessive and Additive) were applied using PLINK software (http://pngu.mgh.harvard.edu/purcell/plink/) to assess the association of SNPs with the risk of alcohol-induced ONFH.

## Abbreviations

NOS3: nitric oxide synthase 3
ABCB1: adenosine triphosphate-binding cassette B1
IL23R: interleukin 23 receptor
PCR: polymerase chain reaction
UEP: unextended mini-sequencing primer
SNPs: single nucleotide polymorphisms
MAF: minor allele frequency
HWE: Hardy - Weinberg equilibrium
OR: Odds ratio
CI: confidence interval
